# Supplementary material for: Learning curve and surgical outcome of robotic assisted colorectal surgery with ERAS program
Source: Sci Rep. 2022 Nov 29;12:20566. doi: 10.1038/s41598-022-24665-w (PMC9709162; doi:10.1038/s41598-022-24665-w)
Supplement: Supplementary file 1 — Supplementary Information 1. [file 41598_2022_24665_MOESM1_ESM.zip › LSC Raw data-Ñ[▒K/051/HS_ñw╝╨░O▒Kñσ.pdf]

正式摘要(電子簽章：00000178625580)

W86 / W86 ☐ 英文標題(注意:無英文姓名-請補病人基本檔!) ☐【讀取】憑證

臺中榮民總醫院

出院病歷摘要

轉入號 : 轉出號 :

[redacted]

(9)入院日期	108/10/16 (2019) 直腸外科 W86 - 152
(10)轉科日期	
(11)出院日期	108/10/24 (2019) 住院天數計 8 日

診斷

- 入院 :- rectal cancer

  = underlying disease
  - hypertension
  - s/p Tubal ligation many years ago
- 出院 :- rectal cancer ,
  Laparoscopic low anterior resection +/- pelvic LN dissection (side to end anastomosis) on 2019/10/17

  = underlying disease
  - hypertension
  - s/p Tubal ligation many years ago

主訴

- bloody stool for 1 month

病史

- This 61 y/o woman had underlying disease of
  - hypertension
  - s/p Tubal ligation many years ago

  This time she suffered bloody stool for 1 month. She also found small caliber for her stool. But she denied any other discomfort. She went to 清泉醫院 for help where colonoscopy showed rectal cancer. Therefore she was admitted for surgical treatment

體檢發現

- Anal inspection:
  Recto digital examination:FINGER TIP
  Sigmoidoscopy:
  Anoscopy:
  Abdomen:
  - General appearance :
    - easy looking , conscious clear , without cardiopulmonary distress
    - Vital signs自動帶入date 體溫(BT) 脈搏(P) 呼吸(R) 血壓(BP) 身高(BH) 體重(BW) BMI
      20191016 36.9℃ 85次/分 18次/分 130/77mmHg 154cm 58kg 24.5
    - Vital signs: B.P. 130 mmHg , P.R. 85 /min , R.R. 18 /min , B.T. 37 ℃
  - HEENT :
    - Eyes: no anemic conjunctiva, no icteric sclera, normal EOM
  - Neck: supple, freely movable, trachea midline,no deformity, no engorged jugular vein , no palpable thyroid or lymph node enlargement
  - Chest:
    - Inspection: Grossly normal, symmetric and fully expansion, no accessory muscle used, no operation scar, no spider angiomata, no gynecomastia
    - Palpation: no palpable mass or nodule, no subcutaneous emphysema, no tenderness

- Ausculation: clear breathing sound
- Heart:
  - Palpation: no thrill, no heave
  - Percussion: normal heart size
  - Ausculation: regular heart beat, normal S1, S2, no gallop, no murmur
- Extremities: No deformity, full ranged of motion without limitation, no clubbing finger or nail cyanosis, no pitting edema, no ulcer or pigmentation, no obvious skin lesion, no varicose vein, no bone, joint or muscle tenderness

CRS CONDITION

- Karnofsky performance status: (100) Normal, no complaints, no evidence of disease
- PE of Abdomen:
- __1. Inspection: ok
- __2. Ausculation: ok
- __3. Palapation: ok
- __4. Percussion: ok
- CEA: 3.77 ng/ml
- Imaging Studies:
- Colonoscope report: nil
- Sono , CT of abdomen and chest Films:pending
- Figures:
  1. colon & rectum

手術日期及方法

- 手術日期及方法20191017 Low anterior resection +/- pelvic LN dissection (side to end anastomosis)

  Laparoscopy

住院治療經過

- After admission, Laparoscopic LAR was performed smootly She was discharged on 10/24

合併症

一般檢查

- 累積報告 :

DATE	TIME	ALB	TP	BIL.T	BIL.D	ALKP	AST	ALT	LDH	CHOL	TG	HDL-C	LDL	UA	NH3	AMY
1081017	1425	4.1		0.6	0.1	80	17	9								

DATE	TIME	WBC	RBC	HGB	HCT	MCV	PLT	BLST	NEUT	BAND	SEG	LYM	MON	EOS	BAS
1081017	1439	14440		11.6	36.9		236		83.6			12.8	3.1	0.3	0.2

DATE	TIME	NA	K	CL	CA	BUN	CREAT	Mg	P	CRP	ESR	PCT	LIPASE	LACTATE
1081017	1425	142	3.5		8.5	7	0.64							

- 放射線報告 :檢驗項目 **:CT ABDOMEN-SURVEY** 報告日期 **:1081022**

  主　訴：
  Result:
  Finding:
  Imaging Report Form for Colorectal Carcinoma (AJCC 8th)
  1. Imaging Date: 20191014
  Imaging Modality, Imaging by
  CT scan
  Imaging Protocol:
  Post IV contrast CT scan of the whole abdomen with 600cc oral contrast showed:
  2. Tumor location / Size
  Location:
  Rectum
  Size:
  Non-measurable
  3. Tumor invasion
  Tumor within colonic or rectal wall
  4. Regional nodal metastasis
  No
  5. Distant metastasis (In this study)
  No or Equivocal
  6. Other findings:
  * Suspicious focal wall thickening of rectum(Se301 Im70). malignancy can not be ruled out

- > Bil. visible lower lungs are clear.
  > Two renal cysts over right kidney
  > Liver,pancreas,spleen,gall bladder,retroperitoneal lymph nodes are not unusual
  Impression:
  Rectal cancer can not be ruled out

特殊檢查

病理報告

- 活體組織切片病理報告 手術日期：**108/10/17** 病理號：**S0836894**

診斷醫師:吳致瑩 報告日期:108/10/23
Pathologic diagnosis:
1. Rectum, Colon, low anterior resection --- a. Adenocarcinoma, moderately differentiated. Tumor invaded to mesocolic soft tissue. The bilateral cut ends and mesocolic lymph nodes (0/16) are free of tumor. b. Tubulovillous adenoma.
2. IMA root, dissection --- Unremarkable fibroadipose tissue, no lymph node seen.
3. Rectum, cut end, distal, resection --- Uninvolved by malignancy.
Ancillary study for diagnosis:
IHC stains for MLH1, MSH2, PMS2 and MSH6 are done on section G. All above markers are preserved.
Prognostic and predictive factor:
1. Tumor size: 2.8x2.8x2 cm.
2. Depth of invasion: mesocolic soft tissue
3. Histologic grade: G2: Moderately differentiated.
4. Angiolymphatic invasion: Not identified.
5. Perineural invasion: Not identified.
6. Tumor deposits (discontinuous extramural extension): Not identified.
7. Surgical Margins: a) Proximal Margin: Uninvolved by invasive carcinoma, distance of tumor from margin: 11.8 cm. b) Distal Margin: Uninvolved by invasive carcinoma, distance of tumor from margin: 4.5 cm. c) Radial or Mesenteric Margin: Uninvolved by invasive carcinoma, distance of tumor from margin: 2 mm.
8. Lymph node status: Uninvolved (0/16).
9. Extranodal involvement: Not identified.
10. Tumor regression grading S/P CCRT: No known presurgical therapy.
11. Type of polyp in which invasive carcinoma arose: Not identified.
12. Additional pathologic findings: Tubulovillous adenoma.
13. Pathological TNM Stage: pT3N0(According to the eighth edition, 2017, American Joint Committee on Cancer Staging Guidelines for Tumors / 2017 CAP guideline).
14. TNM descriptors: Not applicable.

Gross description:
The specimen consists of 1) a segment of colon, 14 cm in length and 4 cm in proximal cut end circumference, 6 cm in distal circumference, and perirectal soft tissue. A gray tan firm annular elevated and centrally ulcerative tumor, 2x2.8x2.8 cm, is noted, 6 cm to the distal cut end. On cross section, the tumor invades to the muscular layer with 20 mm to the radial margin. The both cut ends are free of tumor grossly. A seperated polyp, 0.3x0.2x0.2cm, is noted at mucosa, 7.2cm from proximal margin. 2) IMA root, a piece of tan yellow soft tissue, 4.5x2.9x0.5 cm. 3) distal cut end, a piece of tan mucosa-covered soft tissue, 2x1.5x0.6 cm. Representative part for section: A-B) all of specimen 2 C) all of specimen 3 D) proximal cut end E) distal cut end F-J) tumor K) polyp L-M) lymph nodes.

Note:
1. The interpretation of MMR panel and recommended studies are as below: (1) No loss of nuclear expression of MMR proteins: low probability of microsatellite instability-high (MSI-H) . (2) Loss of nuclear expression of MLH1 and PMS2: testing for methylation of the MLH1 promoter and/or mutation of BRAF is indicated (the presence of a BRAF V600E mutation and/or MLH1 methylation suggests that the tumor is sporadic and germline evaluation is probably not indicated; absence of both MLH1 methylation and of BRAF V600E mutation suggests the possibility of Lynch syndrome, and sequencing and/or large

- deletion/duplication testing of germline MLH1 may be indicated). (3) Loss of nuclear expression of MSH2 and MSH6: high probability of Lynch syndrome (sequencing and/or large deletion/duplication testing of germline MSH2 may be indicated, and, if negative, sequencing and/or large deletion/duplication testing of germline MSH6 may be indicated). (4) Loss of nuclear expression of MSH6 only: high probability of Lynch syndrome (sequencing and/or large deletion/duplication testing of germline MSH6 may be indicated). (5) Loss of nuclear expression of PMS2 only: high probability of Lynch syndrome (sequencing and/or large deletion/duplication testing of germline PMS2 may be indicated). (6) Please note: There are exceptions to the above IHC interpretations. These results should not be considered in isolation, and clinical correlation with genetic counseling is recommended to assess the need for germline testing.
  2. This case has been peer reviewed by two doctors.
  #T-59600_2 #M-81403_2 2 1105 S0000F
  #T-59600_2 #M-82630_2 0 1105 000000
  #T-C4520_2 #M-09183_2 0 1105 000000

其它

出院時情況

- 3.改門診治療

出院指示

藥名	劑量	劑量單位	用法	頻次	數量	數量單位	現況	天數	單位
Ulstop tab 20mg	1	TAB	PO	BID	24	TAB	TH	12	天
Diovan tab 80mg	1	TAB	PO	QD	12	TAB	TH	12	天
Ultracet tab	1	TAB	PO	Q6H	48	TAB	TH	12	天
Mag. Oxide tab	2	TAB	PO	TID	72	TAB	TH	12	天

出院計劃

- 門診日期
  - date on 2019/10/29

主治醫師_2062F 蔣鋒帆 / 住院醫師_5657L 林楙時

正式摘要(電子簽章：**000000178625580)**

W86 / W86

doctorRank:B
